# Refractive error characteristics in patients with congenital blepharoptosis before and after ptosis repair surgery

**DOI:** 10.1186/s12886-016-0351-9

**Published:** 2016-10-08

**Authors:** Ji-Sun Paik, Su-Ah Kim, Shin Hae Park, Suk-Woo Yang

**Affiliations:** 1 Department of Ophthalmology and Visual Science, Seoul St. Mary’s Hospital, College of Medicine, The Catholic University of Korea, 222 Banpo-daero, Seocho-Gu, Seoul, 137-701 Republic of Korea; 2 Deparment of Opthalmology, SamYook Medical Center, Mangwooro-82, Dongdaemoon-Gu, Seoul, Republic of Korea

**Keywords:** Amblyogenesis, Amblyopia, Astigmatism, Congenital ptosis, Refractive error

## Abstract

**Background:**

We examined the effect of surgical repair on the pattern of refractive errors in Korean patients with congenital blepharoptosis.

**Methods:**

We reviewed the clinical records of 54 patients with congenital blepharoptosis who attended our hospital from 2006 to 2012 and underwent a detailed refractive examination before and after ptosis repair surgery. Among them, 21 of the patients whose refractive data was available for both before and after the surgery were included in order to observe the effect of ptosis repair surgery on refractive error characteristics. The astigmatism groups were divided into three subgroups: with-the-rule (WTR), against-the-rule (ATR), and oblique astigmatism (OA). We also evaluated the severity of astigmatism.

**Results:**

Before surgery, the ptotic eyes had more severe astigmatism and a greater percentage of OA than the fellow eyes. The changes in astigmatism magnitude before and after surgery were not significant, but the proportion of subjects with OA increased significantly. In ptotic eyes, amblyopia was found in 14 eyes (20.9 %). 3 eyes (4.5 %) were from solely occlusive visual stimulus deprivation due to severe ptosis, and 11 eyes were from refractive errors. Among refractive errors, amblyogenic astigmatism made up to the largest proportion of patients (8 patients, 11.9 %).

**Conclusions:**

Ptotic eyes had more severe astigmatism and more OA than fellow eyes. Amblyogenic astigmatism was more common in ptotic eyes. A change in astigmatism toward the OA axis was significantly detected after surgery, and that can be possible amblyogenic cause. Therefore, the correction of astigmatism before and after ptosis repair surgery is very important to prevent amblyopia.

## Background

Congenital blepharoptosis is as an eyelid disorder frequently associated with amblyopia, refractive error, anisometropia, and strabismus [1–3]. Patients with congenital ptosis may be at increased risk of amblyopia. Amblyopia can be attributed to refractive errors, occlusion of the visual axis, or associated strabismus. Some authors have stated that stimulus deprivation amblyopia is rare and the head compensation mechanism in humans may countereffect [4]. Amblyopia normally coexists with strabismus and refractive errors, including astigmatism, anisometropia, and ametropia, which can cause amblyopia, regardless of ptosis [4]. However, some authors have reported that amblyopia is closely associated with the severity of ptosis, and that the severity of amblyopia is directly proportional to the severity of ptosis [5, 6].

Until now, no study has investigated the refractive status in Asian children with congenital blepharoptosis. Asian upper eyelids have a characteristic fullness and an absent or less pronounced eyelid crease, or prominent epicanthal folds than Western children [7]. These features may change the refractive characteristics of cornea and hence the refractive status of eye compared to Western individuals. Moreover, after ptosis repair surgery, newly developed vertical elevating power can alter refraction. Although these effects are similar for Asians and Westerners, they might be more severe in Asians due to the thicker eyelid.

In this study reviewed the records of 54 consecutive patients with congenital ptosis to compare the refractive errors between ptotic eyes and fellow eyes and assess the difference in refractive errors before and after ptosis repair surgery. Furthermore, we also evaluated the incidence of amblyopia in ptotic eyes and investigated its cause, assessing whether it originated mainly from the severity of ptosis or that of the refractive errors.

## Methods

### Patients

This study was approved by the Ethics Committee of the Catholic University of Korea (Seoul, Korea) and complied with the tenets of the Declaration of Helsinki for biomedical research involving human subjects. The study protocol and supporting documents were reviewed and approved by our institutional review board. The preoperative and postoperative records of 54 consecutive patients with congenital ptosis, which had been surgically corrected between 2006 and 2012 at the eye clinic of Seoul St. Mary’s Hospital were reviewed using data such as age, sex, degree of ptosis, and refractive errors under cycloplegia.

The inclusion criteria were: unilateral or bilateral congenital ptosis, no other ophthalmic (including strabismus) or systemic disorders, retinoscopic refraction under cycloplegia, and patients older than 4 years and below the age of 20 years. Patients who were younger than 4 years old and who had acquired ptosis, ophthalmologic or systemic disorders were excluded from the study. We also excluded patients with significant strabismus (≥20 prism dioptre) after a single cover uncover test. Previous reports of congenital eyelid ptosis reported that 35 % of congenital ptosis patients had genetic, chromosomal, or neurological conditions [8]. We excluded patients with these conditions, because different systemic conditions can affect refractive errors, and we wanted to chracterize only ptosis-related eyelid effects on refraction. The inclusion criteria were fulfilled by 54 patients. The average patient age (mean ± standard deviation) was 15.09 ± 4.16 years (range, 5–19 years). The male to female ratio was 2.6:1.

### Ophthalmic examination

Routine ophthalmic examinations were performed in all patients. Best corrected visual acuity was determined using a Snellen chart and was noted in decimals; all refraction measurements are expressed in the minus cylinder form for consistency. Palpebral fissure and levator function were measured by the lid excursion method, which was a measurement of the excursion of the upper lid from extreme downgaze to extreme upgaze with the action of the frontalis muscle blocked. Blepharoptosis was classified as mild [marginal reflex distance 1 (MRD 1) ≥ 2 mm], moderate (0 ≤ MRD 1 < 2 mm), or severe (MRD 1 < 0 mm). The MRD1 is a measurement from the central upper eyelid to the pupillary light reflex [9, 10]. Amblyopia was defined as visual acuity <1.0 (20/20), or a 2-fold difference in visual acuity between eyes lines or more difference between 2 eyes while wearing the proper prescription [4, 11].

### Ptosis repair surgery

We performed ptosis repair surgery mainly using the pentagonal sling method of frontalis brow suspension with preserved fascia lata (sling material). All surgeries were performed under general anesthesia with a pentagonal sling method on the upper eyelid. Temporal and nasal eyelid incisions were made, and three incisions (lateral, medial and forehead) were made on the eyebrow. The Wright needle entered the nasal incision above the eyebrow down to the periosteal lining and continued to the back of the orbital septum. Then, the needle was directed from the inner lid and passed along the medial side of the lid incision. Afterwards, the sling material was directed from the inner lid and passed through the inner brow incision. Again, the needle entered from the temporal brow incision and passed through the temporal lid incision. Subsequently, the sling material was pulled up to the lateral part of the eyelid incision. The Wright needle was then directed from the central forehead incision to the lateral and medial brow incisions. A square knot regulated the eyelid margin height in the limbus position and was fixed by a PROLENE® suture. Finally all lid and eyebrow incisions were sutured [12].

### Refractive error examination

Cycloplegic refraction was measured after three administrations of 1 % cyclopentolate and 0.5 % phenylephrine eye drops at 15-min intervals. All procedures were performed using a handheld retinoscope by the same ophthalmologist (corresponding author, Yang) before and 3 and 12 months after ptosis repair surgery. Preoperative refraction was checked in 54 patients to compare the amount of refraction between fellow eyes and ptotic eyes. Preoperative and postoperative refraction in 21 patients was checked to compare for refractive changes before and after ptosis repair surgery in the ptotic eye. Some refractive data 12 months after ptosis surgery were missed, so we can use these data only in power vector analyses.

### Astigmatism

Using standard definitions of astigmatism, when the steeper meridian was close to the vertical meridian (15° to either side of the 90° meridian), it was classified as ‘with-the-rule’ (WTR) astigmatism. When it was close to the horizontal meridian (15° to either side of the 180° meridian), it was classified as ‘against-the-rule’ (ATR) astigmatism. When the steepest and flattest meridians were not close to either side of the vertical or horizontal meridian within the range mentioned above but still maintained a perpendicular orientation to each other, it was referred to as ‘oblique’ astigmatism (OA) [11]. Amblyogenic astigmatism was defined as a < −1.5 dioptre cylinder (DC) or > 1.0 DC difference compared with the opposite eye. We defined ambylogenic astigmatism using our own criteria based on previously reported studies [4, 8]. Anisometric amblyopia was defined as decreased visual acuity because of a refractive difference between eyes of at least 1 D of myopia, 2 D of hyperopia, or 1.5 D of astigmatism [8].

### Power vector analyses

The following equations were used for vector analyses:

$$ \mathrm{J}0 = \mathrm{C}/2 \times \cos\ 2\upalpha $$

And

$$ \mathrm{J}45 = \mathrm{C}/2 \times \sin\ 2\upalpha $$

Where C and α were the minus astigmatism power and the astigmatism axis, respectively, J0 was the orthogonal astigmatism with perpendiculars of 90° and 180°, with a positive value indicating ATR anterior corneal astigmatism, and J45 was the oblique astigmatism at 45° and 135° [13, 14].

### Statistical analysis

All data are expressed as the mean ± standard deviation. Independent sample *t*-tests were used to compare the spherical equivalent refraction (SER) and astigmatism between fellow and ptotic eyes. Chi-square tests were used to compare the frequency of amblyopia and astigmatism type before and after ptosis repair surgery in the ptotic eyes. The paired sample t-test was used for analyzing refractive error before and after ptosis repair surgery. All analyses were performed using SPSS for Windows, ver. 17.0 (SPSS Inc., Chicago, IL, USA). A *p*-value <0.05 was considered to be statistically significant.

## Results

All 108 eyes of the 54 patients were analyzed. The male to female ratio was 2.6:1. Ptosis was bilateral in 13 cases (24.1 %) and unilateral in 41 cases (75.9 %). Table 1 summarizes the demographics of the patients.

**Table 1:** Demographics of included subjects

Characteristic	
Patient (eyes)	54 (108)
Age (years), mean ± SD	15.1 ± 4.2
Sex, M/F ratio	2.6:1
Ptosis type (%)	
Unilateral	41 (75.9 %)
Bilateral	13 (24.1 %)

*SD* standard deviation

Initially, we compared the frequency of amblyopia and astigmatism magnitude between fellow and ptotic eyes; the results are summarized in Table 2. The ptotic eyes had significantly higher prevalence of amblyopia than the fellow eyes (*p* = 0.02, chi-square test), and the magnitude of astigmatism differed significantly between the fellow and ptotic eyes (*p* = 0.02, independent *t*-test). In the astigmatism axis group, no significant differences appeared between the fellow and ptotic eyes.

**Table 2:** Comparison of amblyopia and astigmatic type between normal eyes and ptotic eyes before ptosis repair surgery

	Fellow eye	Ptotic eye	*P* value
Numbers of eyes	41	67	
Amblyopia	2 (4.9 %)	14 (20.9 %)	0.02*
Astigmatism, Mean ± SD (range)	−0.96 ± 1.07 (−4.5 ~ 0)	−1.46 ± 1.30 (−5.25 ~ −0.5)	0.02**
Spherical equivalent refraction (SER)	−1.05 ± 1.89	−1.54 ± 4.09	0.24*
Astigmatic type			0.81*
OA	17 (41.5 %)	32 (47.8 %)	
ATR	3 (7.3 %)	4 (6.0 %)	
WTR	21 (51.2 %)	31 (46.3 %)	

*Chi-Square test, *P* < 0.05 means statistically significant

** Independent t-test, *P* < 0.05 means statistically significant

**Fig. 1:**
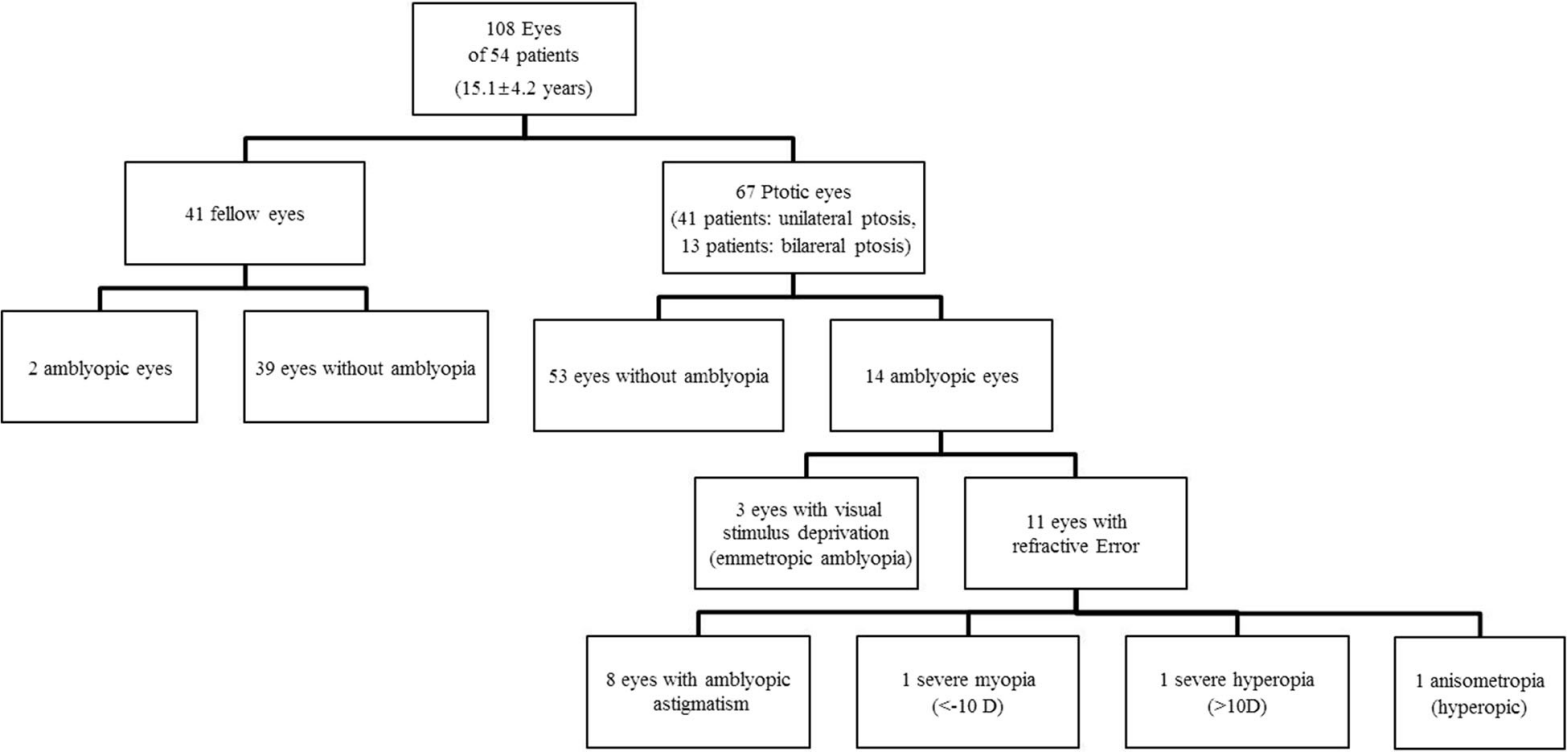
Flowchart of the study (cause of amplyopia)

We compared the clinical and refractive findings between ptotic eyes without amblyopia and ptotic eyes with amblyopia. Table 3 summarizes these results. The two groups did not differ significantly in ptosis severity. The ptotic eyes with amblyopia exhibited greater myopia than the ptotic eyes without amblyopia (*p* = 0.01, chi-square test). The two groups did not differ significantly in terms of astigmatic magnitude, but in the ptotic eyes with amblyopia the frequency of less severe astigmatism (≥ − 1.00 DC; for example, −0.25 DC or −0.75 DC was included)) was significantly higher than in the ptotic eyes without amblyopia (*p* = 0.04, chi-square test). In the ptotic eyes with amblyopia, the frequency of OA was significantly higher than in the ptotic eyes without amblyopia (*p* = 0.03, chi-square test). A comparison of refractive type revealed that ptotic eyes without amblyopia had a significantly higher incidence of emmetropia than did ptotic eyes with amblyopia (*p* = 0.01, chi-square test). We also evaluated the causes of amblyopia (Fig. 1). Out of 14 eyes with amblyopia, visual stimulus deprivation was assumed to be the main cause of amblyopia in three eyes (21 %). In the rest 11 eyes (79 %), refractive error was assumed to be the main cause of amblyopia. 1 eye had severe hyperopia (>10 dioptre) and 1 eye had severe myopia (<−10 dioptre). 8 eyes were amblyogenic astigmatism and among them 5 eyes showed oblique astigmatic axis. Final 1 eye showed anisometropic hyperopia.

**Table 3:** Summary of clinical and refractive findings in ptotic eyes with/without amblyopia in the data from ptotic eyes before ptosis repair surgery

Results for individual ptosis	Ptotic eye without amblyopia	Ptotic eye with amblyopia	*P* value
Number of eyes	53	14	
Ptosis severity			0.18
Mild (MRD1 ≥ 2 mm)	3 (5.7 %)	3 (21.4 %)	
Moderate (0 ≤ MRD < 2 mm)	33 (62.3 %)	7 (50.0 %)	
Severe (MRD1 < 0 mm)	17 (32.0 %)	4 (28.6 %)	
Spherical equivalent refraction (number %)			0.01*
≥ − 1.00 D	42 (79.2 %)	6 (42.9 %)	
< −1.00 D	11 (20.8 %)	8 (57.1 %)	
Astigmatism			
Mean ± SD	−1.37 ± 1.20	−1.92 ± 1.73	0.09
≤ − 1.50 DC (number, %)	22 (41.5 %)	10 (71.4 %)	0.04*
Oblique astigmatism (number, %)	14 (25.9 %)	8 (57.1 %)	0.03*
Refractive type			0.01*
Hyperopia	9	1	
Myopia	11	8	
Emmetropia	33	5	

*MRD1* marginal reflex distance 1

*Chi-Square test, *P* < 0.05 means statistically significant

**Fig. 2:**
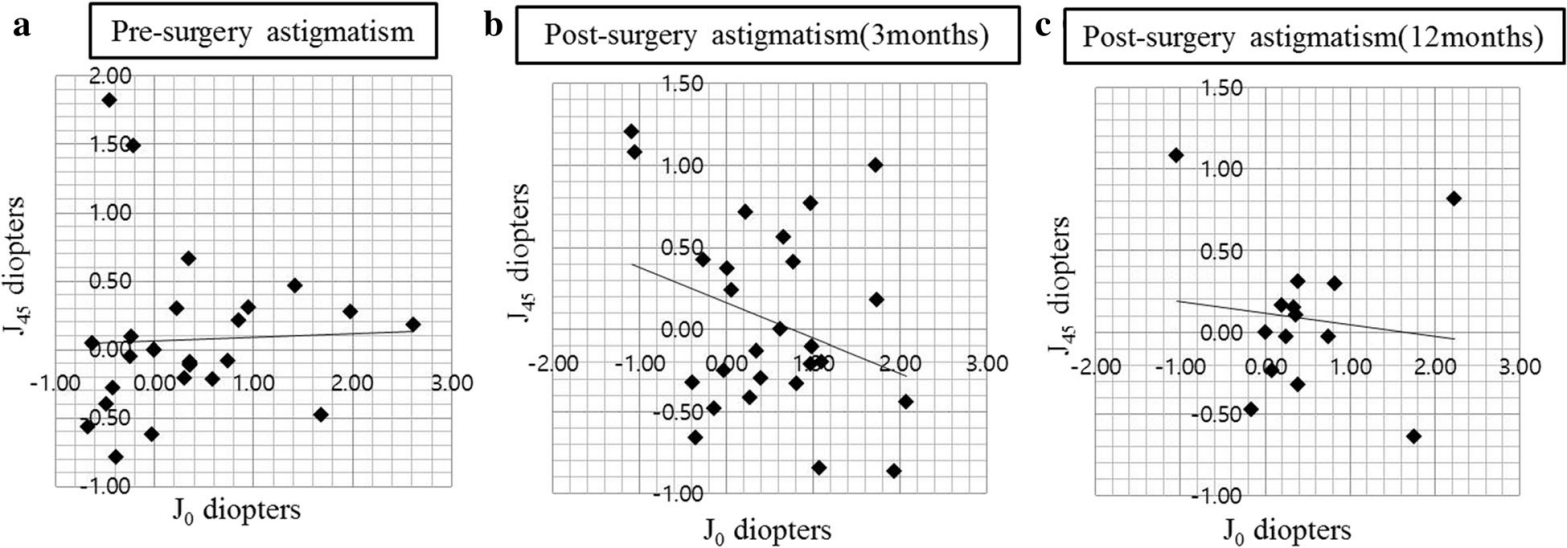
Manifest astigmatism by power vector analysis before surgery (**a**), post-surgery after 3 months (**b**), and post-surgery after 12 months (**c**), There is a distinct change of astigmatic angle axis

**Fig. 3:**
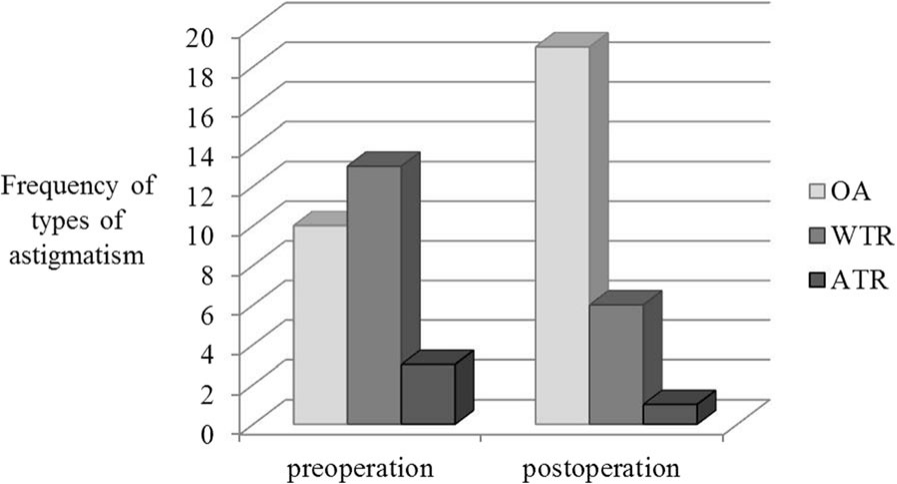
Astigmatic angle axis change before surgery and 3 months after ptosis repair surgery. In Table 4, same results are presented at percentage and there is statistical significance between two groups

Finally, we compared the refractive changes before and after ptosis repair surgery by the paired sample t-test and power vector analysis (Tables 4, 5, and Fig. 2). No significant changes were observed in SER or astigmatic value before and after surgery. However, in terms of the frequency of various types of astigmatism, the occurrence of OA was significantly higher after surgery than before surgery. In Fig. 3, bar graph shows the change in the astigmatic axis before and 3 months after ptosis repair surgery, indicating a change in the astigmatic axis to OA after surgery, which results are also shown in Table 4.

**Table 4:** Comparison of refractive change before and after ptosis repair surgery in the data from refractions before and 3 months after ptosis repair surgery

(Number, 26 eyes)	Preoperation	Postoperation	*P* value
SER (Mean ± SD)	0.65 ± 1.79	0.25 ± 1.94	0.22
Astigmatism (range)	−1.67 ± 1.32 (−5.25 ~ 0)	−1.86 ± 1.16 (−4.25 ~ −0.5)	0.30
Astigmatic type number (%)			0.04*
OA	10 (38.5 %)	19 (73.1 %)	
WTR	13 (50.0 %)	6 (23.1 %)	
ATR	3 (11.5 %)	1 (3.8 %)	
Amblyopia	6 (23.1 %)	9 (34.6 %)^a^	0.37

(Total numbers of eyes were 26 eyes from 21 patients)

*Chi-Square test, *P* < 0.05 means statistically significant

^a^Among postoperation amblyopia, 4 eyes are newly onset amblyopia with amblyogenic astigmatism (1 eye) or oblique astigmatism (3 eye)

**Table 5:** Statistical summary of the distribution of manifest refractive errors before and 3 months and 12 months after ptosis repair surgery

	Before surgery			After surgery 3 months			After surgery 12 months		
	M	J_0_	J_45_	M	J_0_	J_45_	M	J_0_	J_45_
Mean	0.63	0.35	0.07	0.38	0.22	−0.05	0.35	0.46	0.08
SD	1.96	0.83	0.58	1.88	0.87	0.58	2.98	0.79	0.46

## Discussion

The prevalence of amblyopia is estimated at 3.0-3.2 % of the general population [3]. However, the rate among patients with congenital ptosis has been shown to be higher [1, 6, 15]. Previous studies of the causes of amblyopia in the general population reported that approximately one-third of cases are the result of anisometropia, one-third are the result of strabismus, and the remaining third are the result of a combination of both disorders or a form of visual deprivation [11]. Amblyopia caused by visual deprivation seems to be the least frequent subtype based on the relative rarity of the primary causative factors, including infantile cataracts (2–4.5 of every 10,000 births) and childhood ptosis (7.9 per 100,000 births) [11].

The cause of the increased prevalence of amblyopia among patients with congenital ptosis is controversial. Although several authors have argued that the occlusive effect of a ptotic eyelid does not interfere with visual development, subsequent reports have demonstrated that 1.6-12.3 % of patients with a diagnosis of congenital ptosis have amblyopia due exclusively to occlusive stimulus deprivation [3, 6, 11]. Of the 67 ptotic eyes in this report, only 3 eyes (4.5 %) had occlusive stimulus deprivation-type amblyopia. Eleven of the ptotic eyes (16.4 %) with amblyopia were due to refractive errors, including SER, amblyogenic astigmatism, myopia, and hyperopia; among these, amblyogenic astigmatism had the highest incidence, involving 8 eyes (11.9 %).

The incidence of amblyopia in patients with congenital ptosis was 20.9 %, similar to previous reports (range, 14-19 %) [3, 4, 11]. Although there have been a large number of reported cases of amblyopia without any other apparent cause than ptosis [5, 6], in our study amblyopia we found that only 4 eyes with severe ptosis had amblyopia, and three of them do not have refractive errors, and that is mainly from visual stimulus deprivation. Except for these 3 eyes, among other eyes, we did not find any association between the degree of ptosis and amblyopia when we compared ptotic eyes with and without amblyopia according to ptosis severity (Table 3). These findings are similar with those of Beneish et al. [16] and Uğurbaş and Zilelioğlu [17] and contrast with those of Hornblass et al. [15]. Grinpentrog et al. [11] reported that in a cohort study, amblyopia occurred in one in seven children diagnosed with ptosis, and that half of those were the result of eyelid occlusion of the visual axis. Our results differ from those of many reports investigating Western children [4–6, 16, 17]. Maseedupally et al. found that several eyelid morphometry appear to influence corneal shape in primary gaze and horizontal palpebral fissure width and upperlid curvature can affect corneal spherical equivalent with difference between ethnicities [18]. This might be due to anatomical morphometrical differences in Korean children, including more puffy eyelids and a more prominent epicanthal fold than Western children. Our result, that refractive errors have major effect on the development of amblyopia in congenital ptosis (78.5 %), was similar to those of Oral et al. [3] and Thapa R [19] in that they mentioned the cause of amblyopia in congenital ptosis mainly came from refractive errors. They reported that 54 % of amblyopia with congenial ptosis mainly came from refractive errors.

In a report focusing on refractive errors in congenital ptosis, Huo et al. [20] observed that form-deprivation myopia was found more frequently in eyes with unilateral congenital ptosis compared with the opposite eye. In our study, these types of ptotic eyes were not observed. We compared the refractive error, astigmatism, and astigmatic axis between ptotic and fellow eyes. We found that ptotic eyes had significantly more amblyopia and more astigmatism than fellow eyes. We evaluated corneal topography and keratometry examination only in cases with very severe refractive errors, so we cannot use these data in comparing between groups.

Considering refractive changes before and after surgery, Kumar et al. [21] found that the refractive changes after brow suspension surgery were transient and not significant. In our study, the changes in the amount of refraction were not significant, but the changes in angle axis were significant and a relatively large number of cases of OA were observed. In power vector analysis, we also found the change of astigmatic axis after ptosis repair surgery, but it was not statistically significant. That might be originated from the change of lid tightness and corneal curved surface after ptosis repair surgery. Chou et al. [22] reported that a smaller degree of initial OA caused amblyopia compared with orthogonal astigmatism. Changes in the OA axis can mean greater amblyogenic astigmatic changes. Our results show that it is important to check for astigmatic changes after surgery, and that these changes are not transient; they persisted 3 months after ptosis repair surgery.

## Conclusions

We recommend frequent refraction tests to ensure that the best spectacle-corrected visual acuity is obtained. Corneal astigmatism is a major problem in ptotic eyes, and cycloplegic refraction is a useful and inexpensive tool to determine whether astigmatism is regular or irregular in these patients. Moreover, these correction methods are essential both before and after ptosis repair surgery because refraction errors (e.g., astigmatism factors) did not improve after surgery, and the angle of astigmatism after surgery was weighted more toward OA compared to before surgery.

This study was retrospective and therefore might be biased due to the limited recorded information. Relative short term follow-up period is also another limitation. Our data did not include corneal topography and globe axial length. A prospective multicenter-based study including different age and ethnic groups is needed. Our findings suggest that more attention should be paid to the early correction of refractive errors (e.g., astigmatism) before and after surgery to prevent the development or worsening of amblyopia.

## Abbreviations

ATR: Against-the-rule
DC: Dioptre cylinder
MRD1: Marginal reflex distance 1
OA: Oblique astigmatism
SER: Spherical equivalent refraction
WTR: With-the-rule
